# Developmental Effects on Auditory Neural Oscillatory Synchronization Abnormalities in Autism Spectrum Disorder

**DOI:** 10.3389/fnint.2019.00034

**Published:** 2019-07-25

**Authors:** Lisa A. De Stefano, Lauren M. Schmitt, Stormi P. White, Matthew W. Mosconi, John A. Sweeney, Lauren E. Ethridge

**Affiliations:** ^1^Department of Psychology, The University of Oklahoma, Norman, OK, United States; ^2^Division of Developmental and Behavioral Pediatrics, Cincinnati Children’s Hospital Medical Center, Cincinnati, OH, United States; ^3^Department of Pediatrics, Emory University School of Medicine, Marcus Autism Center, Atlanta, GA, United States; ^4^Schiefelbusch Institute for Life Span Studies and Clinical Child Psychology Program, University of Kansas, Lawrence, KS, United States; ^5^Kansas Center for Autism Research and Training (KCART), Kansas City, KS, United States; ^6^Department of Psychiatry and Behavioral Neuroscience, University of Cincinnati, Cincinnati, OH, United States; ^7^Department of Pediatrics, Section on Developmental & Behavioral Pediatrics, University of Oklahoma Health Sciences Center, Oklahoma City, OK, United States

**Keywords:** autism spectrum disorder, EEG, chirp, sensory, development

## Abstract

Previous studies have found alterations in 40 Hz oscillatory activity in response to auditory stimuli in adults with Autism Spectrum Disorder (ASD). The current study sought to examine the specificity and developmental trajectory of these findings by driving the cortex to oscillate at a range of frequencies in both children and adults with and without ASD. Fifteen participants with ASD (3 female, aged 6–23 years) and 15 age-matched controls (4 female, aged 6–25 years) underwent dense-array EEG as they listened to a carrier tone amplitude-modulated by a sinusoid linearly increasing in frequency from 0–100 Hz over 2 s. EEG data were analyzed for inter-trial phase coherence (ITPC) and single-trial power (STP). Older participants with ASD displayed significantly decreased ability to phase-lock to the stimulus in the low gamma frequency range relative to their typically developing (TD) counterparts, while younger ASD and TD did not significantly differ from each other. An interaction between age and diagnosis suggested that TD and ASD also show different developmental trajectories for low gamma power; TD showed a significant decrease in low gamma power with age, while ASD did not. Regardless of age, increased low gamma STP was significantly correlated with increased clinical scores for repetitive behaviors in the ASD group, particularly insistence on sameness. This study contributes to a growing body of evidence supporting alterations in auditory processing in ASD. Older ASD participants showed more pronounced low gamma deficits than younger participants, suggesting an altered developmental trajectory for neural activity contributing to auditory processing deficits that may also be more broadly clinically relevant. Future studies are needed employing a longitudinal approach to confirm findings of this cross-sectional study.

## Introduction

Autism spectrum disorder (ASD) is a highly heritable neurodevelopmental disorder that is diagnosed by behavioral observation of deficits in social communication and the presence of restricted, repetitive behaviors (American Psychiatric Association., 2013). Recently, sensory abnormalities, which may affect up to 90% of individuals with ASD (Leekam et al., 2007), were added to the diagnostic criteria (American Psychiatric Association., 2013). Sensory abnormalities may be among the earliest emerging symptoms (McCormick et al., 2016). However, sensory issues in ASD are highly heterogeneous, with complaints of both hypo- and hypersensitivity, and their underlying neurophysiological mechanisms remain poorly understood.

A reduction in GABAergic inhibitory interneurons, particularly those expressing the protein parvalbumin (PV+), is a common feature in mouse models of ASD (Gogolla et al., 2009) that has been suggested as a potential mechanism for sensory abnormalities in neurodevelopmental disorders. This view has been supported in human post-mortem studies documenting reductions in the number of PV+ interneurons (Hashemi et al., 2016), or in the ratio of PV+ interneurons to other subtypes (Zikopoulous and Barbas, 2013). Brain imaging studies also have implicated PV+ interneurons abnormalities in ASD through findings of reduced neural synchrony (Lajiness-O’Neill et al., 2018). The activity of inhibitory interneurons underlies high frequency beta (12–30 Hz) and gamma (30–80 Hz) oscillations (Whittington et al., 2000) that have been associated with automatic processing of stimulus features during sensory processing, but also higher order cognitive functions (Kaiser and Lutzenberger, 2003). Adolescence has been shown to be a particularly important time in the maturation of beta and gamma band responses (Trevarrow et al., 2019). Additionally, PV+ interneurons’ crucial role in the opening and closing of critical periods of plasticity has led to the assertion that imbalance of excitatory and inhibitory activity may contribute to heterogeneous developmental profiles within ASD, including those associated with sensory processing issues (LeBlanc and Fagiolini, 2011).

Previous findings from studies of cortical oscillations in ASD during sensory tasks have been mixed. For example, Orekhova et al. (2007) used electroencephalography (EEG) to examine the total power of ongoing neural oscillations while children watched soap bubbles or moving fish on a computer screen. Their measure, which was not locked in time to a stimulus, revealed significantly greater power in low gamma frequencies (24.4–44 Hz) in children with ASD than in typically developing (TD) controls. They also reported that greater low gamma power was associated with a greater degree of developmental delay in ASD. Of note, power in higher frequencies decreased with age in TD participants, whereas this was not true for individuals with ASD.

On the other hand, other studies of cortical oscillations have examined baseline-corrected, evoked power during auditory tasks. These studies primarily examine activity evoked by the stimulus, rather than ongoing activity that is re-organized (induced) to respond to the stimulus. Port et al. (2016b) used magnetoencephalography (MEG) to examine neural response to short auditory tones but found no differences between ASD and control groups in evoked gamma power across both children and adult participants. They also examined inter-trial phase coherence (ITPC), a measure of the stability of the response across trials at each frequency. They found significantly greater ITPC in TD than in ASD, a group difference primarily driven by ITPC deficits in adults with ASD. Another study by the same research group used a longitudinal approach to examine evoked gamma power and ITPC with short tones (Port et al., 2016a). Children were recruited between the ages of 6–11 and brought back 2–5 years later. Gamma power differed at both timepoints, with children with ASD exhibiting less evoked power, but ITPC reductions in ASD were only significant at the follow-up visit (Port et al., 2016a). Together, these previous studies of cortical oscillations in ASD document mixed findings, potentially due to methodological differences such as stimulus modality or operationalization of power. Overall, a clear consensus has not been reached regarding alterations to gamma activity in ASD and their developmental changes.

Another approach to studying the relation of gamma band activity to auditory function is to use tones that oscillate at the frequencies of interest. This approach takes advantage of the resonant properties of the cortical interneuron networks in the gamma frequency range to examine basic auditory cortex function. The neural networks responsible for representation of the stimulus should oscillate in time with the stimulus, increasing the signal-to-noise ratio at that frequency and allowing for evaluation of the brain’s sensory response using imaging techniques like MEG or EEG. For example, a 40 Hz steady-state auditory tone would “drive” neural networks to oscillate at 40 Hz, thus synchronizing brain oscillations to the auditory stimuli. Wilson et al. (2007) used a 40 Hz steady state tone to investigate auditory processing during MEG and found that children and adolescents (aged 7–17) with ASD had less evoked 40 Hz gamma power in the left hemisphere but not the right, compared to TD. On the other hand, Edgar et al. (2016) used a 40 Hz steady state paradigm during MEG and found no group differences between children and young adolescents (aged 7–14) with ASD and TD in measures of evoked power or ITPC in either hemisphere. The authors noted that both TD and ASD had a small increase in ITPC with age, suggesting that the networks that synchronize steady-state oscillatory activity may not be fully developed until after adolescence (Edgar et al., 2016).

Previous studies in ASD have examined only a limited number of frequencies, with a focus on 40 Hz as an indicator of gamma response. One alternative would be to use a chirp stimulus, which is an amplitude-modulated tone that increases in modulation frequency from 1 to 100 Hz over the course of 2 s (Artieda et al., 2004). Like the steady-state response, auditory sensory cortex will entrain to a chirp stimulus and oscillate at the same modulation frequency, sharpening the sensory representation of the tone. In Fragile X syndrome (FXS), the leading inherited single-gene cause of ASDs, significantly less ITPC in gamma frequencies were found when using the chirp stimulus during EEG compared to TD controls, and more severe reductions in ITPC were tightly linked to increased background gamma power (Ethridge et al., 2017). These deficits were correlated with not only clinical ratings of sensory processing abnormalities but also clinical measures of social and communication deficits. This suggests that this approach may be useful for identifying alterations in neural oscillations within the auditory cortex in individuals with ASD, and that these abnormalities may be related to broader disorder-relevant symptoms beyond sensory issues.

The current study examined the neural response to the chirp stimulus in ASD, with an emphasis on age-related differences that have not been previously explored over this range of frequencies. We expected that individuals with ASD would demonstrate less ITPC and more gamma, as found in FXS. We also expected these abnormalities would be more pronounced in adulthood, as found when using a steady-state stimulus (Edgar et al., 2016). Additionally, we sought to determine the extent to which abnormalities in high frequency phase-locking and power related to clinical measures in ASD.

## Materials and Methods

### Participants

Fifteen individuals with ASD (*M* age = 12.93, age range = 6–23, *SD* = 5.05; 3 female) and 15 age-matched controls (*M* age = 13.67, age range = 6–25, *SD* = 6.00; 4 female) were recruited through University of Texas Southwestern Medical Center (Table 1). Exclusion criteria included current seizure disorder, traumatic brain injury, non-verbal IQ <60, or use of psychotropic medications with known effects on EEG such as anticonvulsants or sedatives. Participants with ASD met diagnostic criteria using the Autism Diagnostic Observation Schedule (ADOS; Lord et al., 2000), Autism Diagnostic Interview (ADI-R; Rutter et al., 2003b) and expert clinical opinion. Exclusion criteria included the use of non-verbal IQ rather than verbal IQ based on data indicating that individuals with ASD show fewer disorder-related weaknesses in non-verbal abilities (Munson et al., 2008). Parents of ASD participants also completed the Repetitive Behavior Scale – Revised (RBS-R; Lam and Aman, 2007) from which obtained scores were used for correlational analyses. TD participants scored less than or equal to 8 on Social Communication Questionnaire (SCQ; Rutter et al., 2003a), had no known psychiatric illness, and had no first- or second-degree relatives with ASD. All participants completed the Wechsler Abbreviated Scale of Intelligence (WASI; Wechsler, 1999) to estimate IQ. The groups did not differ on non-verbal IQ (see Table 1).

**Table 1:** Demographic information.

Variable	*n*	*M*	*SD*	*t*	*p*
**Age**				0.36	0.72
ASD	15	12.93	5.05		
TD	15	13.67	6.00		
**Age: child**				0.16	0.87
ASD	7	8.86	1.77		
TD	7	8.71	1.50		
**Age: adult**				0.66	0.52
ASD	8	16.5	4.14		
TD	8	18.00	4.90		
**Female**				0.19	0.67
ASD	3				
TD	4				
**PIQ**				0.88	0.39
ASD	11	104.50	16.16		
TD	11	99.18	12.13		
**SCQ**				6.69	<0.01
ASD	12	21.92	5.45		
TD	14	2.86	2.1		


### Procedure

As previously done (Ethridge et al., 2017), participants passively listened to a “chirp” stimulus, a 1000 Hz carrier tone amplitude modulated by a sinusoid that linearly increased in frequency from 0–100 Hz over the course of 2 s. The stimulus was delivered at 65 db via headphones while participants watched a silent movie and underwent dense array EEG. Participants listened to 200 tones separated by an intertrial interval that randomly varied between 1500 and 2000 ms. EEG was continuously sampled at 512 Hz, with a 5th-order Bessel anti-aliasing filter at 200 Hz, using a 128 channel BioSemi ActiveTwo system (BioSemi Inc., Amsterdam, Netherlands) with electrodes placed according to the International 10/10 system (Chatrian, 1985). All sensors were referenced to a monopolar reference feedback loop connecting a driven passive electrode and a common mode sense active electrode, both located on posterior scalp.

### EEG Processing

Raw data were visually inspected offline and bad electrodes were interpolated using spherical spline interpolation in BESA 6.0 (MEGIS Software, Gräfelfing, Germany). No more than 5% of electrodes were interpolated per subject. Data were filtered from 0.5 to 120 Hz (12 and 24 db/octave rolloff; zero-phase) and notch filtered at 60 Hz. Eye, cardiac, and muscle movement artifacts were removed using independent component analysis in EEGLAB 13 (Delorme and Makeig, 2004) for Matlab (The Mathworks, Natick, MA, United States). Data were re-referenced to the average of all electrodes and epoched into 3250 ms trials (-500 ms to 2750 ms), then baseline corrected. Trials with post-preprocessing amplitude ranges greater than 120 μV were removed prior to averaging. The number of valid trials did not differ between groups (ASD *M* = 166.6, *SD* = 27.5; TD *M* = 174, *SD* = 17.18, *t*(28) = 0.88, *p* = 0.38).

Due to our interest in age effects, we classified participants based on neural signatures that have been shown to reflect the maturation of the auditory system (Ponton et al., 2000; Poulsen et al., 2009). This method was chosen instead of classifying based on chronological age due to variability in development during our age range of interest (Sharma et al., 1997; Poulsen et al., 2009), leading to the possibility that different EEG electrodes may reflect the auditory response in adolescents with more adult-like neural activity relative to adolescents with child-like activity. Additionally, due to the potential for positive peaks in children and negative peaks in adults to occur at the same time in the same electrodes (Johnstone et al., 1996; Ceponiene et al., 2002), the use of a grand average containing participants of all ages could attenuate the auditory event-related potential (ERP).

Electroencephalographs were visually examined to determine the valence of the P1-N1-P2 complex, a series of archetypal ERPs that are elicited by auditory stimulation. Each subject’s data was classified as child-like if the N1 showed no clear frontocentral negativity but rather a temporal organization characteristic of immature auditory cortical development (Ponton et al., 2000), or adult-like if separate, frontally located peaks for the P1, N1, and P2 components were discernable. Using this classification system, clear differences were found between the youngest in the sample and the oldest, such that the youngest were always classified as child-like and the oldest were always classified as adult-like. There was overlap between the age groups around adolescence, with one 10-year-old ASD participant and one 11-year-old TD participant classified as having adult-like auditory topography, while the other four 10-year-olds (1 ASD, 3 TD) and the additional 11-year-old (ASD) in the sample were all classified as having child-like auditory topographies and thus analyzed as part of the “child” group. This variation around the age of 10 years is consistent with the literature describing individual differences in maturation of the N1 ERP (Poulsen et al., 2009), and ASD and TD did not differ in number of participants aged 10–11 that were assigned adult-like auditory topography. See Supplementary Figure S1 for a histogram displaying the age distribution and assignment, and Supplementary Figure S2 to compare activity of participants aged 10–11 that were assigned as having child- or adult-like auditory activity.

**FIGURE 1:**
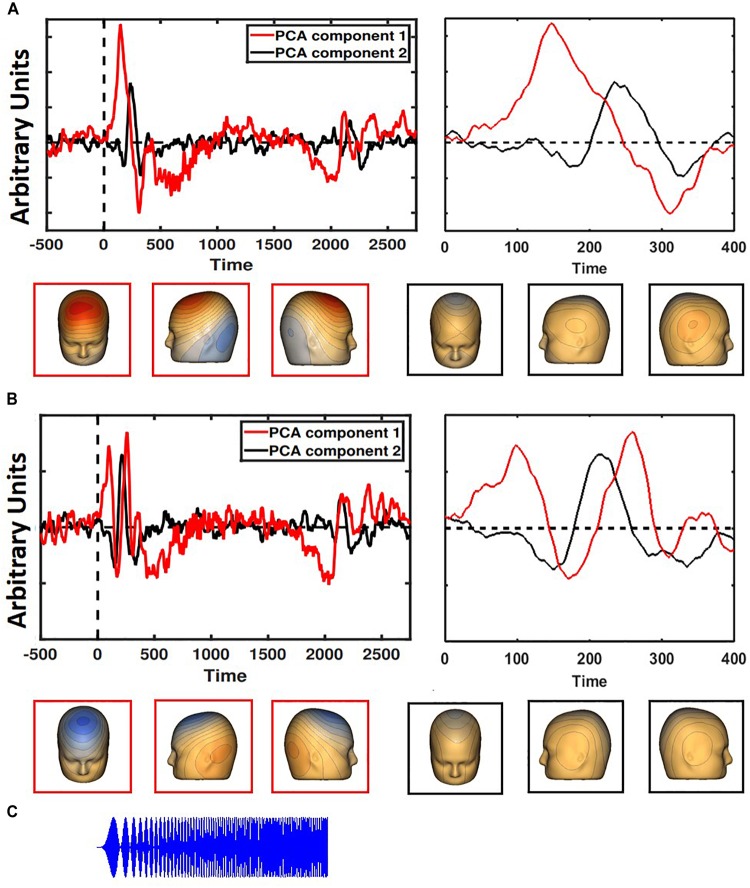
Two PCA components each were generated for children **(A)** and adults **(B)**, with the component accounting for the most variance in red and the second most in black. The entire time series is on the left; a closer look at the first 400 ms is on the right. Child-like and adult-like auditory topographies were assigned based on the lack of a visible N1 (seen in adult-like topography, but not child-like). Representative scalp topographies, below, were taken from the height of the N1 response in adult-like topography (170 ms). Topographies from component 1 are in red; component 2 are in black. **(C)** The stimulus is a two-second long amplitude-modulated tone.

To utilize data from every electrode and ensure accurate localization of auditory cortex, spatial principal components analysis (PCA) was implemented on the grand average ERP (Ethridge et al., 2016, 2017) separately for participants with adult-like and child-like auditory topographies. For both adult-like and child-like responses, the two components that accounted for the most variance were selected (adult-like: 75.4 and 11.4%; child-like: 78.1 and 8.5%; see Figure 1) and the component weights were multiplied by each subject’s average data, summed across electrodes, and divided by the sum of the component weights, reducing the waveforms from one for each electrode to one waveform per component with a known distribution across the scalp. The resultant two waveforms per individual were then weighted in terms of the amount of variance accounted for by each component to leave one virtual waveform on which analyses were conducted.

Time-frequency analyses were performed on PCA-weighted, un-baseline-corrected epoched single-trial data using Morlet wavelets with 1 Hz frequency steps using a linearly increasing cycle length from 1 cycle at the lowest frequency (2 Hz) to 30 cycles at the highest (120 Hz). Inter-trial phase coherence (ITPC), a measure of phase-locking across trials, was calculated to determine the stability of the response, with values closer to 1 indicating higher phase coherence. Single-trial power (STP) was also calculated at each frequency on this PCA-weighted waveform. Raw ITPC values were corrected for trial number by subtracting the critical *r* value for each subject based on individual trial count. ITPC and STP were averaged over trials for each participant and down-sampled to 250 time bins.

In addition to the PCA-weighted measure of STP, STP was calculated for each of the 128 electrode sites and averaged according to hemisphere to examine hemispheric variations in topography. Unlike the PCA-weighted virtual waveform, this unweighted STP measure is not impacted by assignment to child-like or adult-like auditory topography because it utilizes all electrodes equally regardless of auditory topography.

Finally, to further examine stimulus-related oscillatory activity, the data were baseline corrected by dividing the power at each timepoint and frequency by the averaged power in that frequency during the baseline period.

### Statistical Analysis

Point-by-point two-tailed *t*-tests were calculated to examine group differences across the time-frequency matrix for both PCA-weighted and unweighted data. For comparisons made with unweighted data, electrodes were divided evenly into two groups and hemispheric averages were obtained. Time-frequency clustering techniques and Monte Carlo simulations controlled for multiple comparisons; to maintain a family-wise alpha of <0.01, a minimum of three sequential time-bins and three adjacent frequencies were required to be significant at a threshold of <0.05. The final determination of statistical significance was made using 2 (ASD vs. TD) × 2 (child- vs. adult-like) ANOVAs. Pearson correlations examined the relationship between measures of ITPC and STP separately for each group. Due to non-normality of our variables of interest, Spearman’s rho was calculated for all correlations including age or clinical measures. Clinical correlations with RBS-R and ADOS are presented as exploratory with the ASD participants, and thus not corrected for multiple comparisons (14 total clinical variables were tested for correlation with the 3 EEG variables of interest). Analyses of baseline-corrected data are presented in Supplementary Materials.

## Results

### EEG

#### ITPC

**FIGURE 2:**
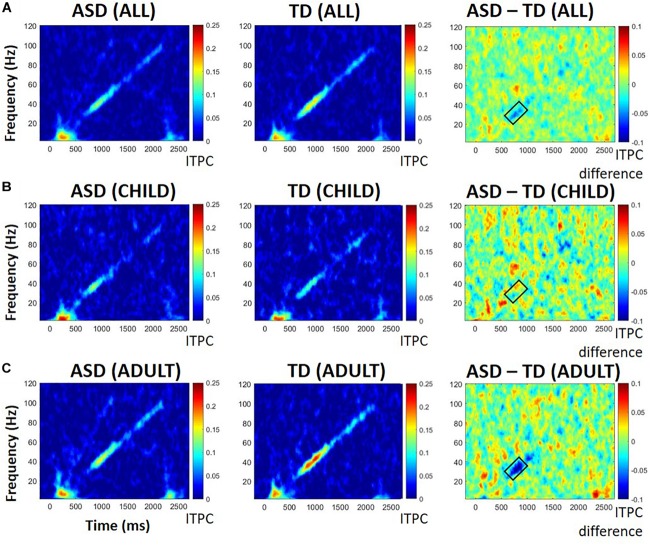
ITPC in all participants **(A)**, those with child-like auditory activity **(B)**, and adult-like auditory activity **(C)**, shown separately for those with ASD (left), TD (middle), and difference between ASD and TD participants (right). Warmer colors in difference plots indicate more ITPC for ASD; cooler colors indicate more ITPC for TD. Black boxes indicate areas of interest representing either significant group differences or significant group by age interactions.

Difference plots identified a cluster of frequencies, 27 Hz to 39 Hz, at which ITPC differed across groups. ANOVA revealed a significant interaction between diagnosis and developmental group, with greater ITPC in TD than ASD among participants with adult-like auditory responses, *F*(1,26) = 5.64, *p* = 0.025, ηp^2^ = 0.178 (see Figure 2). There was a marginal main effect of diagnosis, *F*(1, 26) = 3.21, *p* = 0.08, with ASD having lower ITPC overall relative to TD, and no significant effect of developmental group, *F*(1,26) = 0.13, *p* = 0.72. See Table 2 for means and standard deviations.

**Table 2:** ANOVA results.

	ITPC		
		
Group	Child-like	Adult-like	
ASD	0.09 (0.06)	0.06 (0.02)	**0.07 (0.05)**
TD	0.08 (0.06)	0.14 (0.06)	**0.11 (0.07)**
	**0.09 (0.06)**	**0.09 (0.06)**	
**PCA-weighted STP**			
ASD	40.95 (1.45)	40.43 (1.69)	**40.67 (1.55)**
TD	41.58 (1.76)	38.48 (1.65)	**39.92 (2.29)**
	**41.26 (1.58)**	**39.45 (1.90)**	
**Unweighted STP**			
ASD	40.55 (1.94)	39.85 (1.27)	**40.18 (1.60)**
TD	40.83 (1.45)	37.48 (1.75)	**39.04 (2.33)**
	**40.69 (1.65)**	**38.66 (1.92)**	
**PCA-weighted STP, baseline only**			
ASD	41.00 (1.39)	40.37 (1.64)	**40.67 (1.51)**
TD	41.54 (1.79)	38.44 (1.70)	**39.89 (2.31)**
	**41.27 (1.56)**	**39.41 (1.90)**	


*Bolded values indicate group means at each level.*

#### STP (PCA-Weighted)

**FIGURE 3:**
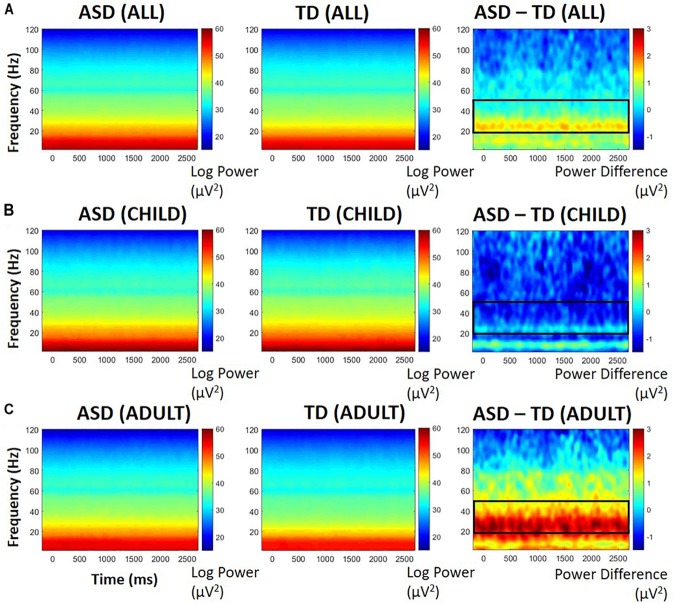
PCA-weighted spectral power in all participants **(A)**, those with child-like auditory activity **(B)**, and adult-like auditory activity **(C)**, shown separately for those with ASD (left), TD (middle), and difference between ASD and TD participants (right). Warmer colors in difference plots indicate more STP for ASD; cooler colors indicate more STP for TD. Black boxes indicate areas of interest representing either significant group differences or significant group by age interactions.

Based on previous research (Ethridge et al., 2017), we were most interested in high frequencies within the gamma range (20 Hz to 100 Hz). Difference plots and point-by-point *t*-tests indicated greater activity between 20 and 50 Hz in ASD participants. The group difference was stable before, during, and after the trial, so statistics were performed on the averaged 20–50 Hz power over the entire epoch. Baseline power in these frequencies was highly correlated with entire-epoch power (*r* = 0.99, *p* < 0.0001). Interestingly, there was an interaction between diagnosis and developmental group, *F*(1,26) = 4.63, *p* = 0.04, ηp^2^ = 0.151: ASD participants with adult-like auditory activity displayed greater STP between 20 and 50 Hz than their TD counterparts (see Figure 3). There was a significant main effect of developmental group, *F*(1,26) = 9.06, *p* = 0.006, ηp^2^ = 0.258, with participants with child-like auditory topographies having greater STP than those with adult-like topographies, but there was no main effect of diagnosis, *F*(1,26) = 1.21, *p* = 0.28.

#### STP (Unweighted)

To further determine whether the significant difference in STP was related specifically to adult-like auditory processing or may be localized differently according to developmental stage, further analysis examined STP at each electrode, rather than relying on PCA weights. Electrodes were then averaged according to their hemisphere (left, right), but no significant differences were found between hemispheres, so the data were collapsed to form one whole-head measure of power between 20 and 50 Hz. This measure was highly correlated with PCA-weighted STP, *r* = 0.92, *p* < 0.0001.

Similar to the PCA-weighted STP results, there was an interaction between diagnosis and developmental group, *F*(1,26) = 5.01, *p* = 0.034, ηp^2^ = 0.162. No main effect of ASD was found, *F*(1,26) = 3.12, *p* = 0.09, but there was a significant main effect of developmental group, *F*(1,26) = 11.73, *p* = 0.002, ηp^2^ = 0.311. ASD participants with adult-like auditory activity displayed greater STP between 20 and 50 Hz than adult-like TD participants. Participants with child-like activity did not differ between diagnosis groups, suggesting that localization was not contributing to the interaction effect.

### Correlations

#### STP and ITPC

**FIGURE 4:**
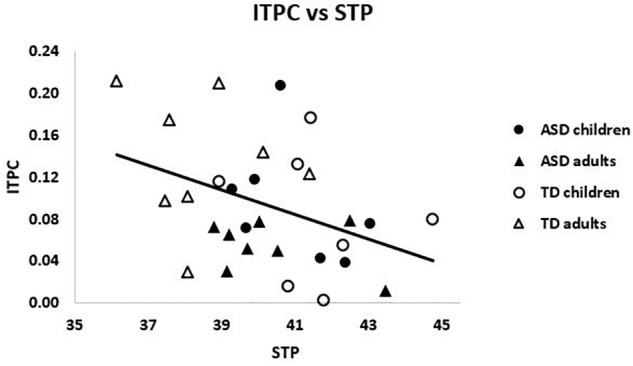
ITPC and weighted STP were negatively related across the sample, *r* = –0.39, *p* = 0.035.

There was a significant negative relationship across the entire sample between PCA-weighted STP and ITPC, *r* = -0.39, *p* = 0.035 (see Figure 4). That is, higher STP was related to decreased ability to synchronize activity with the chirp stimulus, suggesting that increased gamma neural noise decreases the signal-to-noise ratio of auditory cortex. However, neither group reached significance on its own (|*r*| ’s < 0.23, *p*’s > 0.1).

#### Relationships With Age

**FIGURE 5:**
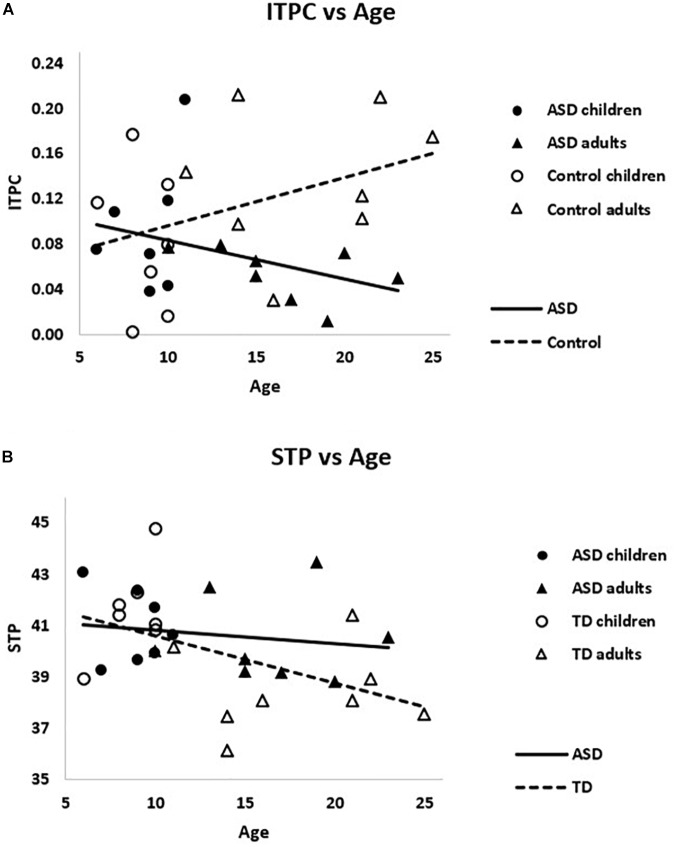
**(A)** The correlations between ITPC and age were significantly different for ASD and TD, *Z* = 1.90 *p* = 0.03. **(B)** Weighted STP was significantly negatively correlated with age in TD (dashed line), *r_s_* = –0.55, *p* = 0.02, but not ASD (solid line), *r_s_* = –0.22, *p* > 0.4.

Within ASD alone, there was a trending relationship between ITPC and age, *r_s_* = -0.39, *p* = 0.15, such that older subjects had reduced ITPC. The opposite pattern emerged in TD, *r_s_* = 0.35, *p* = 0.2, such that older subjects had increased ITPC. These correlations were significantly different between groups, *Z* = 1.90 *p* = 0.03, suggesting divergent patterns of ITPC and age in ASD and TD (see Figure 5A).

Considering gamma power, PCA-weighted STP and age were negatively correlated across the sample, *r_s_* = -0.42, *p* = 0.02, such that higher STP was associated with younger ages. This relationship remained significant when TD were examined alone, *r_s_* = -0.55, *p* = 0.02, but not within ASD alone, *r_s_* = -0.23, *p* > 0.40 (see Figure 5B). Though the difference between these correlations was not significant, *Z* = 0.94, *p* > 0.15.

#### Clinical Correlations

**FIGURE 6:**
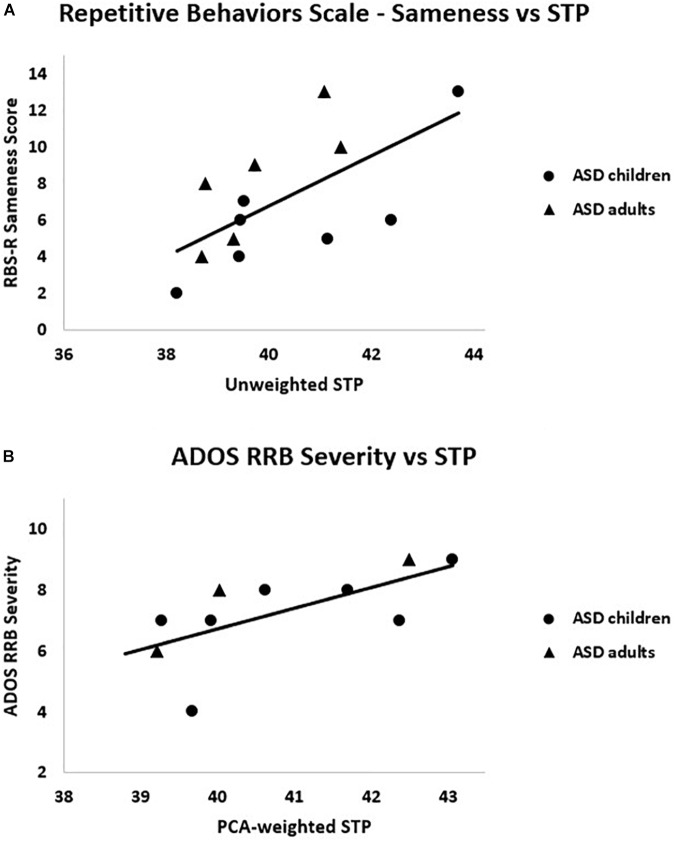
Within the ASD group, **(A)** RBS-R Sameness subscale scores were positively correlated with unweighted STP, *r_s_* = 0.67, *p* = 0.01; (**B**) ADOS RRB severity scores were positively correlated with PCA-weighted STP, *r_s_* = 0.81, *p* = 0.005.

Clinical correlations of interest are presented in Table 3. Unweighted STP and the RBS-R Sameness subscale scores were significantly correlated, *r_s_* = 0.67, *p* = 0.013. Additionally, PCA-weighted STP related to ADOS Restricted and Repetitive Behavior (RRB) severity scores, *r_s_* = 0.81, *p* = 0.005 (see Figure 6). That is, higher STP was related to more severe RRBs in ASD. IQ, SCQ and ADOS total scores were not significantly correlated with any EEG measure, nor was ITPC correlated with any clinical measure (*r*’s < 0.4, *p*’s > 0.3).

**Table 3:** Clinical correlations.

	Clinical Measure			
	
	RBS-R Sameness Subscale		ADOS Repetitive and Restricted Behavior	
	
EEG Measure	*n*	rho	*n*	rho
PCA-weighted STP, 20–50 Hz	13	0.44^∧^	10	0.81^∗^
Unweighted STP, 20–50 Hz	13	0.67^∗^	10	0.60^∧^


*^∧^*p* < 0.15; ^∗^*p* < 0.05.*

## Discussion

In the current study, we document new findings regarding age-related neural responses to sensory stimuli in ASD using a stimulus that entrained auditory cortex to linearly increasing frequencies. First, ASD participants with adult-like auditory topographies showed less phase locking than their TD counterparts across high beta/low gamma frequencies (27–39 Hz). These results suggest that the inhibitory network function that determines the ability to phase-lock to an oscillatory stimulus is abnormal in adults with ASD, but not necessarily children with ASD. Second, ASD participants with adult-like auditory topographies showed greater STP between 20–50 Hz than age-matched TD participants. This increased background gamma power has been characterized as neural “noise” that may interfere with the ability to efficiently process incoming stimulation (Ethridge et al., 2017; Goswami et al., 2019). Last, increased STP appeared to be selectively related to the severity of restricted, repetitive behaviors in ASD, suggesting their relevance to the pathology of ASD. Together, our findings provide novel evidence of disrupted gamma activity in adolescents/adults with ASD but not children, suggesting certain abnormalities in neural oscillations may not emerge until later in development.

### EEG Measures

Our ITPC results are consistent with findings showing significantly less ITPC in adults with ASD than TD adults (Port et al., 2016b) as well as previous studies documenting no group differences between children with ASD and their TD counterparts (Edgar et al., 2016; Port et al., 2016b). In addition, we documented that ITPC reduces with increasing age in the ASD group but increases with age in TD group, consistent with previous studies reporting ITPC increases throughout development (Cho et al., 2015; Edgar et al., 2016). These findings suggest ITPC is relatively intact during childhood in individuals with ASD, but differences relative to control begin to emerge in adolescence/early adulthood in ASD. Though some of the previous studies indicated absence of group differences between ASD and TD in childhood may be due, in part, to inability to capture a steady state response, we were able to acquire viable measures of ITPC in children in both groups, suggesting our finding of similar ITPC between groups during childhood reflects a developmental effect rather than a floor effect from reduced signal.

Whereas TD adults had significantly less low gamma STP than children, STP remained relatively constant among children and adults with ASD. This age-related decrease in STP found in our TD sample is in line with findings of decreases in gamma activity during development (Tierney et al., 2013). Of note, this finding held both when PCA weights were applied to the data to examine electrodes responsive to the auditory stimulus, as well as when all electrodes were included equally in the analysis. This rules out the possibility that assignment to adult-like or child-like auditory cortex impacted our estimation of spectral power.

Our baseline-corrected analyses (see Supplementary Materials) found no significant differences between groups, as expected. Because the chirp response is largely created by phase resetting and not power increases, our findings support the hypothesis that power differences in ongoing (and not necessarily stimulus-related) oscillations distort the signal-to-noise ratio in ASD and impair stimulus processing. In all, our findings indicate abnormal neural activity in response to the chirp tone that appears to emerge in adolescence/adulthood, and thus may be related to dysmaturation of neural circuitry occurring over this developmental period.

### Relationships With Clinical Measures

We importantly document the relationship between our EEG auditory measures and ASD symptomology. As our findings were selective to RRBs, this suggests disrupted neural mechanisms underlying our STP/ITPC findings also may contribute to RRBs. It is important to note that our correlations with RBS-R Insistence on Sameness and ADOS RRB suggest that these relationships were not necessarily driven by sensory issues, as only a portion of the ADOS RRB score may be accounted for by sensory symptoms. Further, RBS-R Insistence on Sameness reflects difficulty dealing with change and preference for routines. Thus, STP/ITPC abnormalities may be a broader reflection of behavioral dysfunction in ASD. Altogether, these results indicate that the prospective decreased neural signal to noise ratio suggested by our STP/ITPC findings has functional consequences that may extend beyond sensory systems.

### Potential Neurophysiological Mechanisms

The current study contributes to a growing literature that suggests abnormalities in neural development in ASD. Gamma waves are generated through recurrent connections between GABAergic inhibitory interneurons and excitatory pyramidal cells (Whittington et al., 2000). Animal models suggest fewer inhibitory interneurons in ASD (Gogolla et al., 2009); studies of human children using MR spectroscopy suggest decreased GABA in auditory cortex in ASD (Gaetz et al., 2014). More cortical GABA has been related to more gamma ITPC in TD children, but this relationship was not found in children with ASD (Port et al., 2016b). However, GABA quantity was unrelated to ITPC in adults with or without ASD, leading the authors to suggest that a certain GABA concentration may be required for the typical development of local circuits responsible for gamma coherence (Port et al., 2016b). This could possibly explain our finding of reduced ITPC in only adolescents/adults with ASD: alterations to ITPC may be emergent based upon GABA quantity during development. Altogether, reductions in inhibitory interneurons and GABA availability are potential mechanisms by which phase-locking and gamma power abnormalities could occur.

Another possible mechanism by which altered developmental trajectories could occur is through a lack of synaptic pruning, particularly within the auditory cortex. Hutsler and Zhang (2010) showed that temporal lobe dendritic spine densities were greater in a small post-mortem sample of ASD relative to TD. Because auditory cortex synapses undergo pruning throughout childhood and into early adolescence (Huttenlocher and Dabholkar, 1997), a failure of this process could contribute to the differential ITPC/STP results we observed between children and adolescents/adults in the current sample. Together, decreased inhibitory tone and increased excitation onto pyramidal neurons could underlie the significant negative relationship between STP (increased) and ITPC (reduced) found in this study. Individual variations in synaptic pruning could lead to the heterogeneous complaints of auditory hypo- and hyper-sensitivity found in ASD. Translation of these findings to rodent models of ASD may provide additional insight on neural mechanisms and novel treatment options that target specific symptoms, as well as periods of plasticity. Promising work is currently underway in the FXS *fmr1* knockout mouse, which also shows increased gamma power and deficits in phase-locking to a chirp stimulus (Lovelace et al., 2018); these gamma power abnormalities may also be responsive to pharmaceutical intervention (Sinclair et al., 2017).

### Limitations

There are certain limitations of the present study. Only a moderate number of participants were tested, and while the use of the chirp stimulus with a similarly sized sample of FXS patients provided robust group differences (Ethridge et al., 2017), a larger sample is necessary to confirm trending age-related findings as well as to further capture and parse individual differences due to the heterogeneity intrinsic to ASD. We are particularly limited by the low number of participants at each age (see Supplementary Figure S2), which may mask age-related effects at the tails of our age distribution. Future studies are needed to determine the extent to which our EEG findings relate to clinical ratings of sensory hyper-sensitivity as found in FXS. Additionally, our results speak to a developmental abnormality that cannot be fully explored in a cross-sectional nature. A longitudinal examination would be warranted to examine individual changes in gamma activity from childhood through adolescence. Further, our study is limited in that it used only auditory stimulation, which may not generalize to other sensory modalities. Another possible limitation is our method of STP analysis that was based on relevance to prior studies in FXS (Ethridge et al., 2016, 2017), however, as other approaches are available (Edgar et al., 2015a,b; Port et al., 2016a). Future studies are needed using both methods within a larger sample.

## Conclusion

This study extends previous research on auditory processing in ASD by documenting reduced neural entrainment to a novel auditory stimulus in older participants, accompanied by increased gamma power. The reduced ability to synchronize neural activity to the chirp in adolescent and adult but not child ASD participants suggests an altered developmental trajectory. The related lack of age-related decrease in gamma STP in ASD provides further evidence of dysmaturation of neural circuits within sensory cortex. The appearance of these oscillatory deficits later in development suggests that late childhood/early adolescence may be a critical period for synaptic pruning related to both sensory and behavioral abnormalities and that treatments targeted at preventing this dysmaturation process may be most effective prior to early adolescence. Measures of repetitive behavior correlated with gamma STP, suggesting the clinical relevance of EEG findings may extend beyond sensory processing in individuals with ASD. Together, our findings provide evidence for age-related disruptions in neural oscillations neural signature that has the potential to inform future pharmaceutical and behavioral interventions, particularly those aimed at determining critical developmental windows for treatment efficacy.

## Data Availability

The datasets used and/or analyzed during the current study available from the corresponding author on reasonable request.

## Ethics Statement

All participants provided written informed consent (caregiver with assent or individual consent as appropriate) prior to participation, as approved by the University of Texas – Southwestern Institutional Review Board.

## Author Contributions

LDS analyzed and interpreted the data, and wrote the manuscript. LS aided in manuscript preparation and conducted clinical assessments. SW conducted clinical assessments. MM and JS were critical to study design and recruitment. LE oversaw EEG data collection and contributed to all aspects of the research process, most significantly in data interpretation and manuscript preparation.

## Conflict of Interest Statement

LE consults to OVID Therapeutics and Fulcrum Pharmaceuticals. The remaining authors declare that the research was conducted in the absence of any commercial or financial relationships that could be construed as a potential conflict of interest.

## Abbreviations

ASD: Autism spectrum disorders
Db: decibels
EEG: electroencephalography
ERP: event-related potential
FXS: Fragile X Syndrome
GABA: gamma-aminobutyric acid
Hz: Hertz
ICA: independent component analysis
IQ: intelligence quotient
ITPC: inter-trial phase coherence
MEG: magnetoencephalography
Ms: milliseconds
PCA: principal component analysis
PV+: parvalbumin positive
RRB: restricted and repetitive behavior
SCQ: Social Communication Questionnaire
STP: single trial power
