# Supplementary material for: Developmental Effects on Auditory Neural Oscillatory Synchronization Abnormalities in Autism Spectrum Disorder
Source: Front Integr Neurosci. 2019 Jul 25;13:34. doi: 10.3389/fnint.2019.00034 (PMC6670023; doi:10.3389/fnint.2019.00034)
Supplement: Supplementary file 1 [file Data_Sheet_1.docx]

Supplementary Material

# Supplementary Data

To further examine stimulus-related oscillatory activity, the data were baseline corrected by dividing the power at each timepoint and frequency the averaged power in that frequency during the baseline period. Difference plots and point-by-point t-tests identified a time period between 730-1050 ms post-stimulus onset during which activity between 40-45 Hz differed based on developmental group, *F*(1, 26) = 21.37, *p* < .0001 (see Supplementary Figure 3). Participants with adult-like auditory responses had increased activity relative to those with child-like auditory responses. There was no main effect of diagnosis, *F*(1, 26) = 0.00, *p* = .96, or interaction between diagnosis and developmental group, *F*(1, 26) = 1.61, *p* = .22.

# Supplementary Figures and Tables

## Supplementary Figure


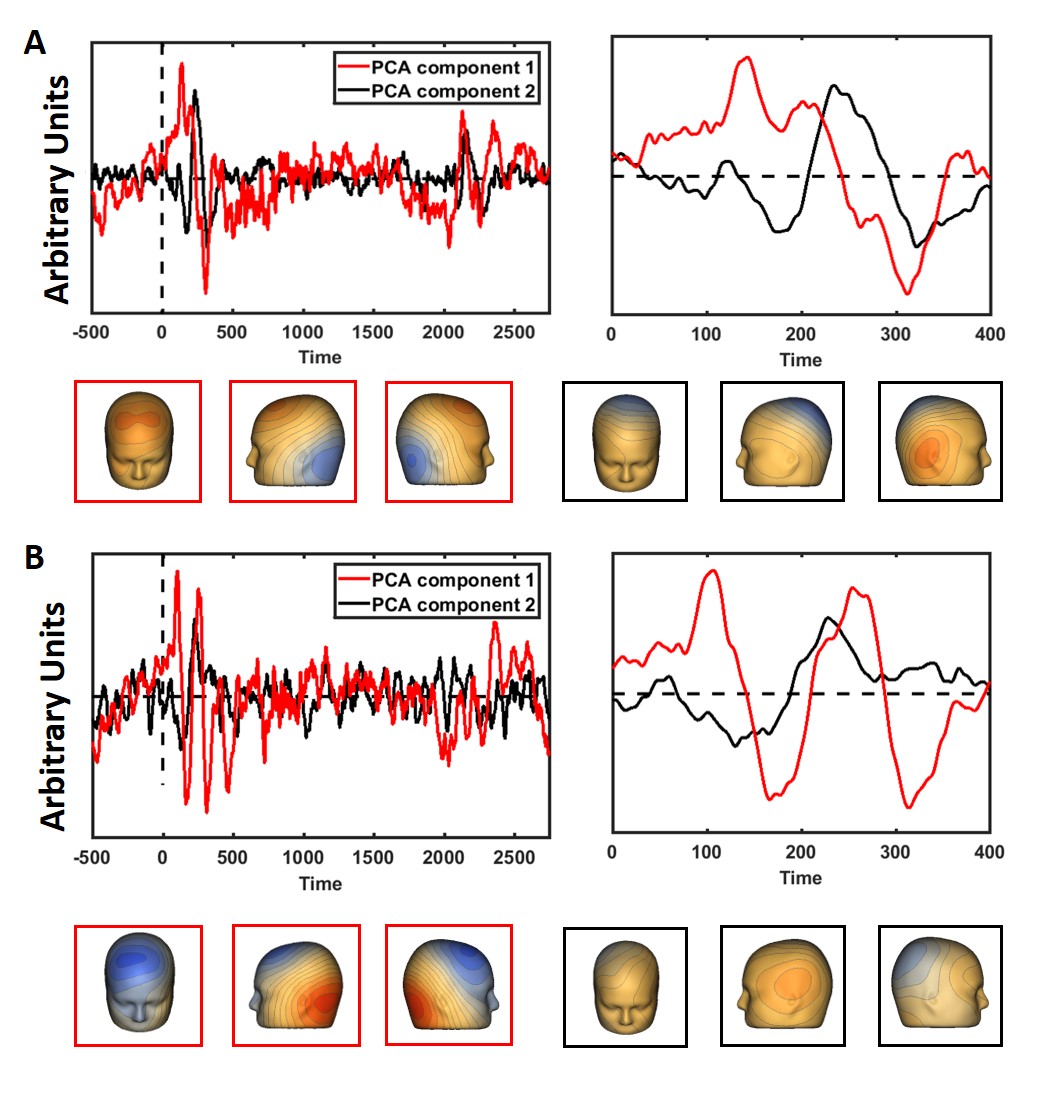


**Supplementary Figure 1.** PCA components for the entire epoch on the left and a closer look at the first 400 ms on the right. Displayed separately for **(A)** five 10-year-olds (2 ASD, 3 TD) and one 11-year-old (ASD) that were deemed to have child-like auditory activity, (**B**) one 10-year old (ASD) and one 11-year-old (TD) that were deemed to have adult-like auditory activity. The component accounting for the most variance in red and the second most in black. Child-like and adult-like auditory topographies were assigned based on the lack of a visible N1 (seen in adult-like topography, but not child-like). Representative scalp topographies, below each set, were taken from the height of the N1 response in adult-like topography (170 ms). Topographies from component 1 are in red; component 2 are in black.


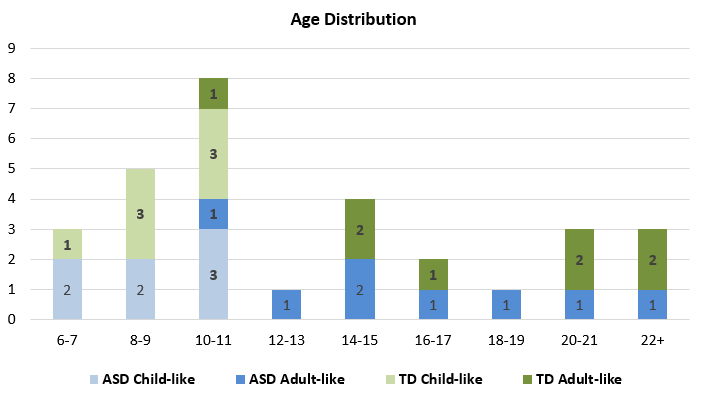


**Supplementary Figure 2.** Histogram showing the number of participants in two-year bins with ASD in blue and TD in green. Lighter colors indicate participants with child-like auditory activity; darker colors indicate adult-like auditory activity.


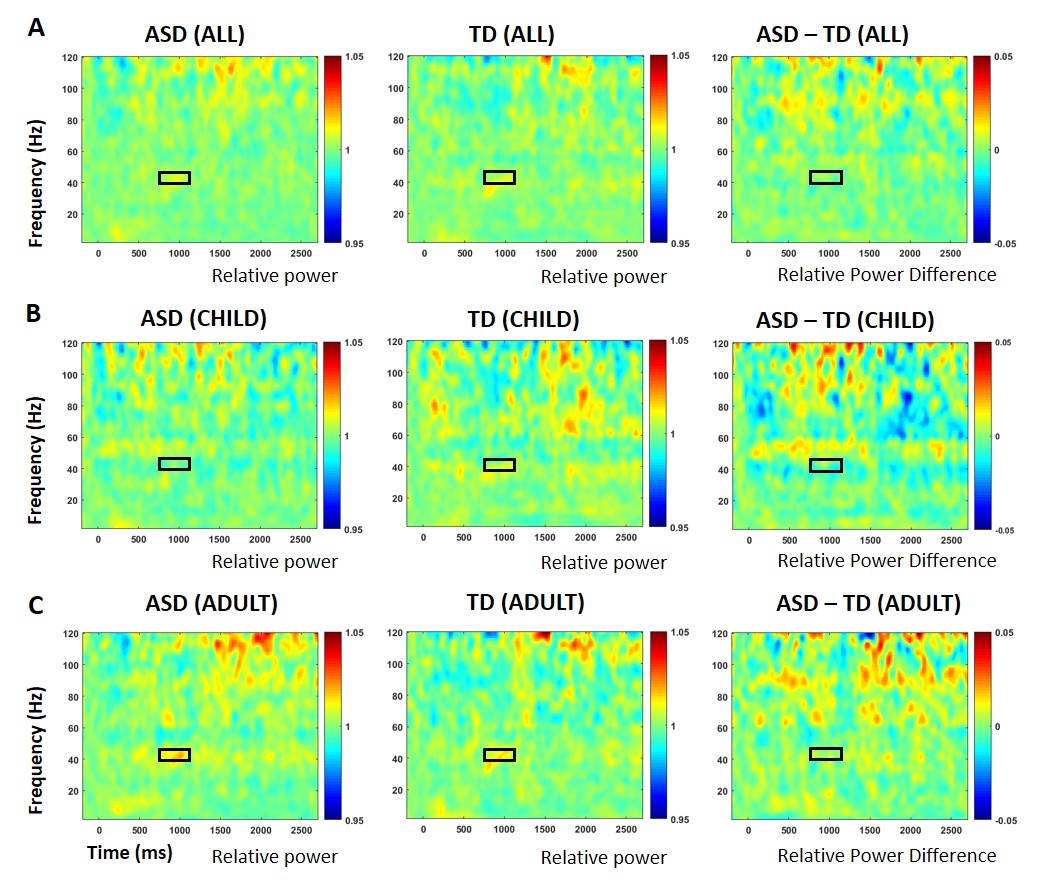


**Supplementary Figure 3.** PCA-weighted spectral power, as a ratio of baseline power in the same frequency for all participants (top row), those with child-like auditory activity (middle row), and adult-like auditory activity (bottom row), shown separately for those with ASD (left), TD (middle), and difference between ASD and TD participants (right). Warmer colors in difference plots indicate more STP for ASD; cooler colors indicate more STP for TD. Black boxes indicate areas of interest representing significant differences.
